# Rethinking violence prevention in elderly and special needs care: requirements for digital solutions

**DOI:** 10.3389/fpsyt.2026.1843725

**Published:** 2026-08-25

**Authors:** Miriam Kappe, Johannes Schobel

**Affiliations:** DigiHealth Institute, Neu-Ulm University of Applied Sciences, Neu-Ulm, Germany

**Keywords:** documentation, elderly care, qualitative research, requirements, special needs care, standardization, violence assessment, workplace violence

## Abstract

**Background:**

The lack of specific guidelines for reporting and documenting violent behavior in elderly and special needs care makes it difficult to compile accurate statistics on incidents. Even though initial violence prevention strategies already exist, a comprehensive, centralized, and process-oriented approach to violence prevention is still missing and often demanded by domain experts. In this context, standardization could help improve visibility of the interrelationships and the problem itself.

**Aims:**

The main focus is to gain a deeper understanding of the processes involved in reporting and handling incidents of violence. Requirements for the digital support of violence protection concepts are identified that help implementing digital applications.

**Methods:**

This manuscripts follows a qualitative approach, where 18 semi-structured interviews were analyzed according the qualitative content analysis by Kuckartz. All interviewees were violence protection officers and managers of elderly and special needs care institutions.

**Results:**

The study identifies various phases in the process of documenting violent incidents that could be supported and automated by digital applications. The main focus is on documenting the incident itself and logging the entire incident handling process. Requirements for a digital application to handle these processes are outlined by this manuscript. Interviewees demand for the implementation of such applications and see them as an opportunity to standardize procedures and to generate and analyze statistics on incidents. This will ultimately lead to more targeted strategies for addressing and preventing violence.

**Conclusion:**

This manuscript provides the foundation for developing digital applications to support violence prevention concepts in elderly and special needs care institutions. Standardization in this domain can improve the management of such incidents for all parties involved. This will also lead to improvements in overall care and working conditions.

## 1 Introduction

Aggression or violence from clients and relatives is a commonly cited and growing source of stress among care personal in Germany (1, 2). This includes behavior that result in physical, psychological, sexual, or financial harm (3). In light of high turnover rates and an increasing willingness to change jobs, this issue deserves high attention (1). At the organizational level, various measures can be implemented to prevent such incidents. These include, for example, the use of electronic health records and notifications regarding individuals at potentially high risk (4). Complementary, systematically reporting incidents of violence serve the structured documentation and can also be viewed as a preventive measure in itself (5).

Existing literature from Germany focuses on environments other than elderly and special needs care (6–9). These works also revealed that there is no standardized approach that would allow incidents to be objectively and transparently assessed, often resulting in an under-reporting of violent incidents. Internationally, the topic of “workplace violence in the healthcare sector” has attracted significant interest and has led countries such as Italy, Sweden, Denmark, and many others to develop nation-wide strategies for reporting such incidents and protecting victims (5). In Germany, there are still few guidelines in this domain, which is a structural barrier for reporting (5). Consequently, it is up to the institutions themselves to develop reporting processes and guidelines.

In addition to training and support services for workplace violence, reporting policies and procedures are needed to deal with under-reporting (10). Fast and easy-to-use reporting systems, feedback following the reporting process, and support from management and supervisors are considered factors that promote the latter (11). At organizational level, this means treating this issue as a management responsibility (12). Furthermore organizational and management interventions should be considered in conjunction with community interventions so awareness of incidents of workplace violence in healthcare is raised (10). This requires the implementation of guidelines as well as monitoring and reporting structures, which allow detailed documentation (12, 13). Standardized assessment tools serve this purpose (14). The collected data can also be used to identify specific measures and conduct risk analyses (13).

Considering potential long-term effects, standardization and digital support of violence protection concepts could contribute to the quality of care for those affected. This manuscript addresses the following research question (**Box 1**):

**BOX 1. Requirements for Digital Documentation of Violence**

What are necessary requirements for developing a digital reporting and documentation system for incidents of violence in elderly and special needs care to ensure an objective, measurable, and transparent representation of post-incident management?

To answer this research question, semi-structured interviews were conducted to identify the processing phases, key requirements for a digital application to document and handle incidents, as well as requirements for an applicable assessment scheme. This manuscript outlines key factors for standardizing the documentation and handling of incidents of workplace violence in elderly and special needs care institutions.

## 2 Materials and methods

This manuscript builds on findings derived from interviews from the publication “Violence Prevention and Incident Documentation in Elderly and Special Needs Care Institutions in Southern Germany: Results from Semi-Structured Interviews” (15) and uses the same data set. However, this manuscript analyzes different aspects and responses of the conducted interviews.

In this context, a practice-oriented research design was applied with the purpose to gain a deeper understanding of existing user needs (16), current procedures for handling incidents of violence, and to assess requirements for a digital application that can be effectively applied in practice.

To get insights into commonly used documentation and handling strategies a qualitative study design was applied. Semi-structured guideline-based interviews were conducted according to the recommendations of Kallio et al. (17). In addition to demographic information, the following topics were included: (1) Incident Handling, (2) Incident Assessment, and (3) Application Requirements. To ensure that the wording was precise and easy to understand, the interview guide was refined iteratively. It was not distributed prior to the interviews. During the interviews, follow-up questions were asked to ensure a better understanding of the responses if required.

The study was conducted and analyzed by the main author as part of her doctoral research. She is a research associate and, drawing on her background in healthcare management, focuses on topics in workplace research. The second author is research professor at Neu-Ulm University of Applied Sciences focusing on “digital medicine and care”. The research project is funded by the Federal Ministry of Research, Technology, and Space (BMFTR). With the exception of one participant, all interviewees were previously unknown to the researchers.

The interviews took place between June and August 2025. After audio recording, the interviews were transcribed and anonymized. Qualitative content analysis according to Kuckartz was applied to evaluate the data (18). For Data analysis the ATLAS.ti software was used. This method was used to systematically categorize the qualitative interview data. The process was carried out iteratively. In the first step, the interview transcripts were read in their entirety, and passages relevant to answering the research question were highlighted. Content categories were then established. The interview questions served as an initial deductive model that defined main categories. The material was coded, and the text passages grouped under the main categories were analyzed again to inductively generate sub-codes. After discussion within the team, it was decided to define “Involved Stakeholders” as a separate main category, since they differ in terms of the functions and data provided and have a corresponding influence on the resulting application. All interview transcripts were analyzed again using the final coding system. The codes developed are listed in **Table 1**.

**Table 1:** Category system for the qualitative analysis.

Code	Sub-code
Descriptive Information	Institution Size: Number of Employees
	Institution Size: Capacity
	Function
	Is there a policy for reporting violent incidents?
Involved Stakeholders	Primary Users
	Communication Contacts
	Secondary Units
Reporting and Documentation Process (Incident Handling)	Case Reporting
	Case Documentation
	Immediate Feedback
	Case Clarification
	Defining Responsibilities
	Development of actionable Strategies
	Victim Support
	Perpetrator Support
	Rehabilitation in Cases of false Suspicion
	Evaluation and Data Management
	Hazard Assessment and Risk Analysis
	Self-Reflection by Employees
Reporting Form (Incident Assessment)	Immediate danger
	Consequences of Violence
	Types of Violence
	Direction/Level of Violence
	Employees’ Qualification
	Contextual Data
	Criminal Offense
	Intent
	Subjective Perception
	Perpetrator’s Perspective
	Severity
	Recurring Incidents
General Requirements (Application Requirements)	Anonymous Reporting
	Documentation/Checklist-Style Form
	Simple Language
	Intuitive Usability
	Notification/Reminder Function
	Case Evaluation
	Statistical Analyses
	Partially Automated Process
	Mobile Reporting Process
	Internal Communication
	External Exchange Communication
	External Case Reporting
	Reminders and Messages
	Status Updates
	Self-Reflection Instruments
	Generation of Accident Reports
	APIs for different Departments
	System Compatibility
	Traceability (Logging)
	Different User Roles

The ethical review of the research protocol was conducted by the Joint Ethics Committee of Bavarian Universities (GEHBa^1^) under vote GEHBa-202501-V-279. All interview participants were fully informed about the study protocol and signed an informed consent form.

Participants were asked about the requirements for an assessment scheme for documenting incidents and the requirements for a digital application for documentation and handling incidents of violence. Since case handling varies depending on the categorization and severity of the incident, information on incident assessment was also collected. **Table 2** shows anexcerptoftheinterview- guideline translatedfromGerman. The original quotations used and their translations can be found in the **Supplementary Material**.

**Table 2:** Excerpt from the interview guide, translated from German.

Topic	Question
Sample Description	What is your job title?
Sample Description	How big is your institution?
Sample Description	What is your area of specialization?
Documentation	What is the purpose of reporting incidents of violence at your institution?
Documentation	Who reports and documents incidents of violence at your institution?
Incident Handling	What steps are taken after incidents of violence are reported?
Incident Handling	In what format is the data collected?
Incident Handling	Who uses the collected data afterward?
Incident Handling	Is there a clearly defined procedure that can be reviewed?
Incident Assessment	Does your institution use a standardized assessment framework when documenting incidents of violence?
Incident Assessment	How is the severity determined?
Incident Assessment	Who is involved in determining the severity level?
Incident Assessment	Who is responsible?
Incident Assessment	How are incidents of violence handled going forward?
Incident Assessment	What requirements do you have for an incident assessment scheme for violent incidents?
Incident Assessment	What factors should be included in an assessment framework for incidents of violence?
Application Requirements	What steps must an application for recording incidents of violence include?

Participants could choose between attending the interview in person or virtually via Zoom. All of the participants opted for virtual participation. Only the interviewer and the participants were present during the interviews. The duration of the interviews ranged from 37 min to 68 min. Data saturation was determined in accordance with the recommendations of Francis et al. Since the analysis of the current situation revealed significant differences between participants in elderly care and those in special needs care, respondents were analyzed separately (19). After conducting five interviews in each group, we checked whether the interviews revealed additional codes. Interviews continued until at least two interviews revealed no new codes. This occurred after five interviews for the elderly care sample and after nine interviews for the special needs care sample.

### 2.1 Participants

Interviewees were recruited through the KompIGA^2^ project network. Additionally an official list of special needs assistance institutions in the state of Baden-Württemberg, Germany, could be used to identify relevant institutions. 18 process-owners in violence protection (i.e. violence protection officers, project employees, de-escalation trainers, institution managers) of 16 elderly and special needs care institutions were interviewed. The researchers were already familiar with one of the interviewees prior to the interviews; all others were recruited as described below. The interviewee acquisition was carried out through direct outreach. A purposeful sampling method was applied. Individuals who are responsible for developing violence prevention concepts are the persons who have the most experience dealing with violence and boundary violations. First contact was made by phone. The research project was presented and contact details were provided. Participants who were interested could volunteer to take part in the interview. A detailed study briefing was conducted to describe that this is a funded research project. The goal of developing a standardized approach to documenting and processing cases of violence was explained. All individuals who expressed interest in the study information participated in the interview. All information regarding data collection, processing, and storage was provided to the participants in written form prior to the interviews. The interview guide was not provided prior to the interview. All participants signed an informed consent form.

Most interviewees were violence protection officers. Additionally de-escalation trainers and institution managers were included, if these were the only people responsible for the topic. One person was a violence protection representative from a large association. In sum, 18 experts from 16 different institutions participated. Seven persons work in elderly care and ten in special needs care institutions. One person works in the headquarters of an association, representing elderly and special needs care institutions with approx. 80,000 clients.

This research is regionally limited to southern Germany. Provided quotes have been translated into English. An overview of the participant sample can be seen in **Table 3**.

**Table 3:** Study participants.

ID	Profession	Primary focus	# Employees	# Clients
ID 1	Organizational Development	Elderly Care	1,500	1,200
ID 2	Project Development Violence Prevention Officer	Elderly Care	220	800
ID 3	Institution Manager	Elderly Care	45	35
ID 4	Head of Professional Development	Elderly Care	3,600	8,700
ID 5	Prevention and Violence Protection Officer	Elderly Care	2,900	6,000
ID 6	Home and Care Service Manager	Elderly Care	34	30
ID 7	Employee Central Quality Management	Elderly Care	600	600
ID 8	Quality and Complaints Management Officer	Special Needs Care	1,600	3,000
ID 9	Consulting and Service, Prevention Specialist	Special Needs Care	5,000	5,200
ID 10	Representative for Women’s- and Violence Protection (Association)	Elderly- and Special Needs Care	37,000	80,000
ID 11	Psychological Specialist and De-escalation Trainer	Special Needs Care	1,300	1,300
ID 12	Religious Care and Violence Prevention	Special Needs Care	600	600
ID 13	Employee Protection and De-escalation Trainer	Special Needs Care	180	200
ID 14	Specialist Service for Prevention and Crisis Intervention	Special Needs Care	2,000	5,000
ID 15	Violence Prevention Officer	Special Needs Care	3,000	3,000
ID 16	Prevention, Violence Prevention, and Sex Education	Special Needs Care	5,200	4,500
ID 17	Violence Protection Officer	Special Needs Care	200	80
ID 18	Admissions Management and Violence Protection Officers	Special Needs Care	1,600	3,000

## 3 Results

This section summarizes important aspects of the conducted interviews related to the overall process of documenting, handling, and archiving violent incidents in institutions of elderly and special needs care. First, insights into relevant and involved stakeholder are presented. Second, a generic reporting and documentation process, derived from the conducted interviews is given. Third, results are outlined with respect to the reporting. Finally, requirements derived from the interviews for developing a digital reporting and documentation system are presented.

### 3.1 Involved stakeholders

Various stakeholders are involved in reporting and handling incidents of violence. These differ in their role within the process and are divided into three different groups: primary users, communication contacts, and secondary units.

- Primary Users: Stakeholders, who are also primary users within the process. Often, they are persons working in the respective institutions. They differ depending on the involved activities within the process. Often, their view on particular information varies (i.e., depending on their role or the current activity).
- Communication Contacts feed information into the process and are notified about the current status. Usually, they are not directly working on a specific case. The type, content, and scope of the information they receive are defined and controlled by the respective process owner.
- Secondary Units are both internal and external parties that receive information about a specific incident, which then may lead to further activities. These are no longer part of incident handling, but may be direct consequences.

**Table 4** lists all the persons involved. Interviews revealed, that these vary depending on the institution and the cases. Particularly in organizations without an established violence protection concept, only few stakeholders were identified. Interviewees mentioned different approaches on who should report incidents and how. For example, while some organizations expected all employees to be able to report incidents, others left the documented reporting based on employee statements to management. It became evident, particularly with regard to secondary units, that clear statements can currently be made only about particularly serious incidents, since there is a requirement to document and externally report such incidents.

**Table 4:** Involved stakeholder groups.

Primary users	Communication contacts	Secondary units
Internal Stakeholders: • Clients. • Employees. • Deescalation Trainer. • Specialist Service. • Religious Care. • Violence Protection Officer. • Manager. • Executive Management. • Division Management.	Internal Stakeholders: • Public Relations • Employees’ Council. External Stakeholders: • Doctor. • Police. • Legal Guardians/Parents. • Witnesses. • Public Persecutor’s Office.	Internal Stakeholders: • Occupational Safety. • Division Managers’ Meeting. • Qualification. • Human Resources Management External Stakeholders: • Superior Association. • Youth Welfare Office. • (German) Employer’s Liability Insurance Association. • Service Providers (District of Swabia).

### 3.2 Reporting and documentation process

**Figure 1:**
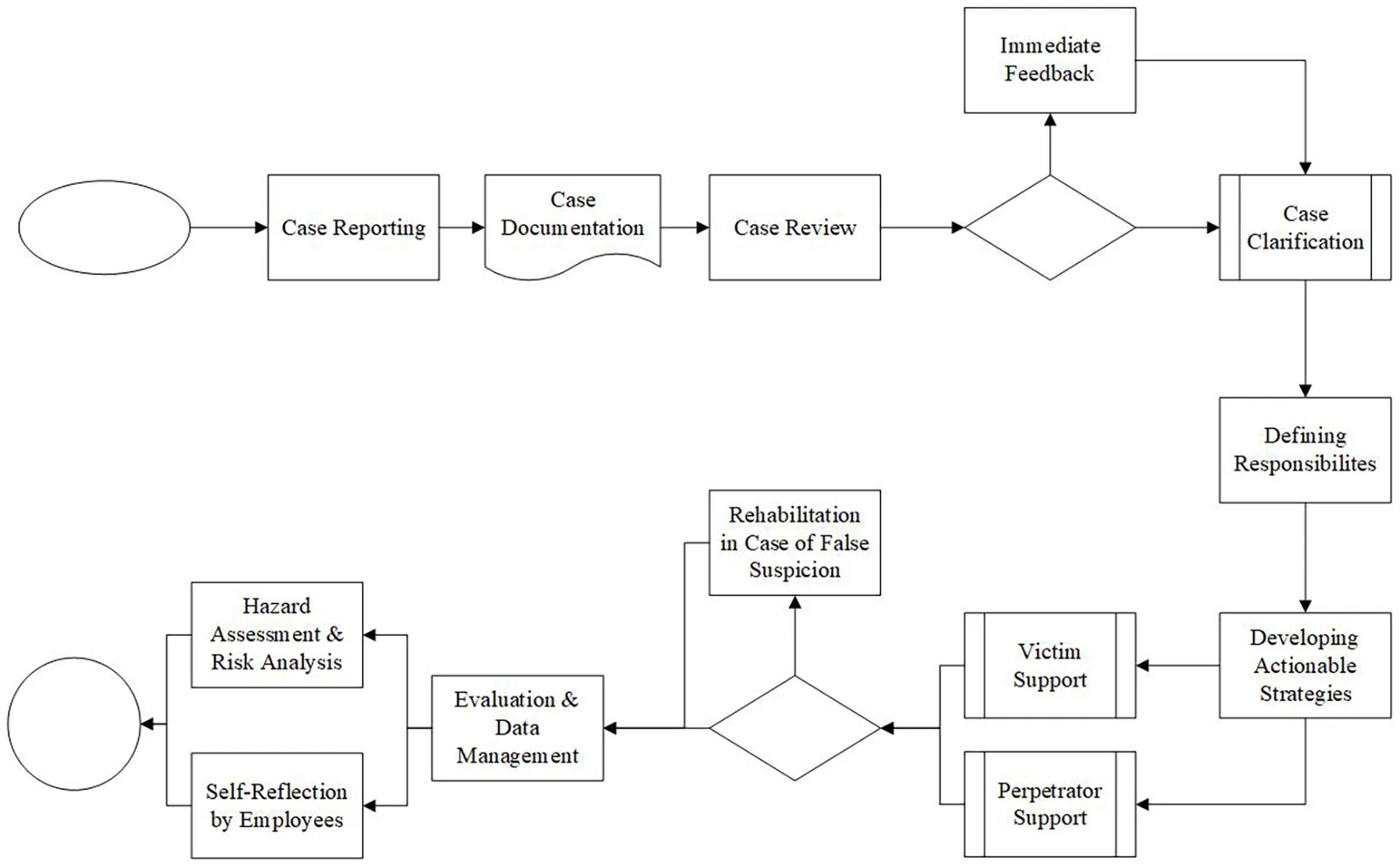
Flowchart for handling incidents of violence.

Participants were asked which activities generally exist in violence protection to get a better understanding of the context of the process. It is also used to identify what information is relevant and how it could be reported and made accessible to others. Note that the answers from participants differed, however, a generic process could be derived (i.e., containing the most important activities mentioned by the interviewees). This generic *as-is* process is illustrated in **Figure 1**.

The first step is case reporting. Currently, the responsible persons are informed in an unstructured way through various channels (i.e., telephone call, paper-based reporting form). This may result in significant delays and misunderstandings. Person 13 stated:

“Either I get a call when I’m on site, or I receive an email. [ … ] I also have to read the documentation [in the client file] that the employees wrote down. This is most likely quite difficult, as I often [haven’t the access rights and] can’t read all required information. If the employee doesn’t check a field in the form, I can’t see the information. That is revealed during personal conversation. An incident may occur, but I only find out about it weeks or months later. So that’s definitely an issue.”

(ID 13, personal communication, author’s translation)

Interviews revealed that the case documentation can be optimized in most institutions. When using a reporting form, it must be supplemented by several additional inquiries from the violence protection officers. There are many media breaks and poor digital accessibility. Person 7 stated:

“[ … ] we have checklists available [ … ]. You can access and print them out yourself.”

(ID 7, personal communication, author’s translation)

After an incident is reported, immediate feedback should be provided by the responsible manager. Person 2 for example stated:

“I give everyone feedback, preferably immediately, to let them know that I have heard them.”

(ID 2, personal communication, author’s translation)

Next, the case clarification follows. In elderly and special needs care, there are clients who are unable to talk about incidents due to limited communication skills. In addition, it is not always clear, especially in people with dementia, whether statements refer to the most recent or to prior events. Further obstacles to clarification may include a lack of observations and evidence. If there is only suspicion, the only thing that can be done is to observe the situation. Interviewees described this task as quite challenging and complicated. Next, all parties involved are interviewed. Person 4 stated:

“The emergency response team decides which other people need to be involved in this case. Is it required to involve the violence protection officer? Is senior management required in the form of regional management? Does it need to be escalated to the management or the execution board?”

(ID 4, personal communication, author’s translation)

The permissions and the level of detail of the information differ per institution. Participants agreed that the individuals involved in handling the case (i.e., part of a convened emergency response team) must have the ability to continuously review the progress and decide on (counter)measures. Person 15 explained:

“It would be great if you continue with the entire protocol there, if you could actually enter the responsibilities, who talks to whom and how, then everything would be in one place. Data protection would be complied with. You wouldn’t have to send it back and forth via email.”

(ID 15, personal communication, author’s translation)

Members of the emergency response team vary depending on the institution and the severity of the case. With the exception of clients, all primary users (see **Table 4**) may be involved in the emergency response team. However, as a rule of thumb, experts explained that mainly senior managers and violence protection managers, as well as religious caregivers or specialist services are usually part of this team.

Further, internal communication involves transmitting of data to collection points. These may be users themselves, or they may receive anonymous information as secondary unit (i.e., public relations, employee representatives,…). The incident data is discussed anonymously, for example in regular division manager meetings. Occupational safety can use the data on risk and hazard assessment and the human resources department must be informed about incidents when employees are involved.

External exchange communication is also necessary at various points in the process (e.g., case clarification). In the event of imminent danger, police or emergency services must be alerted immediately. In less acute cases, general medical care is included. In addition, external psychosocial care or counseling services can be utilized. In serious cases, consultation with a lawyer or the public prosecutor’s office may also be necessary and caregivers or relatives must be informed.

When enough information about an incident has been collected, defining responsibilities follows:

“Clear responsibilities must be assigned. [ … ] It is important that all communication is traceable.”

(ID 15, personal communication, author’s translation)

To achieve this, one needs to assign a person who can provide direct support and is in charge of managing this incident. Depending on the type and severity, different roles must be involved (see **Table 4**). Responsibilities are then assigned among the team members. These responsibilities also include documentation duties, handling internal and external communication, and providing information that is shared among stakeholders.

Next, interviewees mentioned the development of actionable strategies and counter measures to deal with an incident. Again, this is highly dependent on various factors (i.e., involved people, severity,…).

“First, there is a direct conversation [with the reporting person] where we discuss the next steps. Then it depends very much on the individual case. [ … ] In one case, supervision is demanded, in another, counseling, and sometimes very quick access to probationary sessions is required [ … ].”

(ID 2, personal communication, author’s translation)

Victim support must be clarified and carried out. The protective function is the main focus here. This accompanies the entire process. Interviewees clarified:

“Cases are directly reported to me. And then it’s a matter of handling the cases. The highest priority is always protecting those entrusted to our care or our employees.”

(ID 5, personal communication, author’s translation)

It is about providing direct care for physical and mental injuries. The physical distance from the suspect may also play an important role. External assistance (i.e., counseling services, crisis intervention, psychological support) will be organized as needed. Even if no support is needed at first, there must be a dedicated contact point through whom possible measures can be initiated.

In order to deal with and counteract lasting trauma, some of the institutions conduct regular moderated interviews and provide therapeutic support when necessary. Study participants revealed the importance of such feedback and information. The measures mentioned may include probationary (therapeutic) sessions, the use of external counseling, supervision, moderated ethical case discussions (involving religious care or psychological services), or labor law measures. Measures are also necessary in case of recurring incidents. This also triggers reviewing older cases and may result in relocating clients to other residential groups.

When providing support for victims, there is also a need for Perpetrator support. Especially in special needs care, perpetrators are usually also protected persons, clients, or employees themselves.

“The perpetrator’s perspective must also be taken into account, because we are usually responsible for the perpetrator as well.”

(ID 11, personal communication, author’s translation)

Person 11 added:

“They [perpetrators] are often in distress and unaware, but simply acting within their own boundaries and possibilities, sometimes going too far.”

(ID 11, personal communication, author’s translation)

Sometimes, the rehabilitation of perpetrators must be planned in cases of false suspicion. It must be carried out jointly as a team. Person 6 stated:

“We always talk to those affected. [ … ] Legal aspects must also be examined. This may be difficult,because on the one hand there is the accusation of violence, but on the other hand it could also be defamation.”

(ID 6, personal communication, author’s translation)

Some interviewees stated that also external case reporting must be taken into account. When employees are affected, the (German) Employer’s Liability Insurance Association must be informed. Some associations also work with external consulting partners in some cases. They compile overviews for different metrics (i.e., types of violence, severity of the threat, injuries, police reports, etc.). In particular, there are special regulations in child and youth welfare services as well, according to which the care home supervisory authority must be notified in the event of serious incidents of violence. Person 16 explained:

“When it comes to minors, i.e., those who are not of legal age, we also have a duty to inform the youth welfare office according to certain criteria.”

(ID 16, personal communication, author’s translation)

After handling incidents, most institutions follow with an evaluation and data management phase. Person 18 stated:

“We also hold annual evaluation meetings with all management teams to review statistics and assess measures for the entire institution [ … ], with the aim to develop new measures at the corporate level.”

(ID 18, personal communication, author’s translation)

The goal of this phase is to optimize existing violence protection concepts. Management, de-escalation trainers, and violence protection officers create anonymized overviews of incidents that can be used for further evaluation. The latter are carried out regularly to identify general trends and derive new preventive measures. Additionally, the case handling itself is evaluated. Person 15 stated:

“We review each case two to three months after it has been resolved. We are not interested in the current status, but rather at how the reporting process worked. Can we identify any problems and improve the process?”

(ID 15, personal communication, author’s translation)

After handling the incident, transferring the data into hazard assessment and risk management may be useful. These are carried out at regular intervals in most institutions by the occupational safety department. Anonymized incident data can be used to assess the potential risk to clients, visitors, and employees. Person 18 stated:

“It is important to consider the spatial situation. What is the staffing situation? What is the professional competence of the employees involved in an incident? How are they being trained? How are they being supported by the specialist service? Then you also have to take into account what is currently happening in their environment. For example, Christmas or any other major annual celebrations, when the residents’ expectations and excitement increase. [ … ] Tension rises, and with it the possibility that certain situations will escalate.”

(ID 18, personal communication, author’s translation)

Finally, self-reflection by employees was often mentioned. Interviewees emphasized, that this phase is not about blaming employees for inappropriate behavior, but rather on questioning actions in the context of existing power relations and boundary violations associated with working with clients. Person 2 stated:

“[The topic of violence] is still not discussed openly enough. It’s there. When you talk to people, they all think, yes, we’re good. We’re already doing a lot. And suddenly, during the conversation, I realize, ah, there’s some self-reflection going on here, that people are asking themselves, yes, but I’m also applying violence.”

(ID 2, personal communication, author’s translation)

This involves both empathic consideration of the client’s situation and reflection on one’s own attitude.

“If I am not aware of my trauma, then of course I will constantly be hurt and experience violence, or things will escalate. And if I am aware, I can protect myself accordingly.”

(ID 13, personal communication, author’s translation)

### 3.3 Reporting forms

The development of a transparent and objective form that enables the automatic classification of violent incidents into a reproducible severity rating appears to be a sensitive topic. However, the interviewees found it difficult to decide on a common ground and implement such regulations, since the perception of violence is considered to be highly individual, and the definition is accordingly based on individual assessment. Nevertheless, scalability and the distinction between minor, moderate, and serious incidents were identified as urgent and important (15). The following requirements should be considered when developing a reporting form for a digital application.

The description of incidents includes contextual data about the location (where) and time (when) an incident happened. Structural conditions, such as power relations, asymmetric communication, the age of the involved individuals, the setting, (overwhelming) working conditions, time pressure, structural factors, and general tolerance to boundary violations can be important indicators, why an incident may have happened. Describing the qualifications of the involved employees were also frequently mentioned during interviews. This is especially important when employees are suspected to be directly involved in an incident (i.e., as perpetrator or victim). Person 15 stated:

“It makes a difference, if it is a student or an experienced professional who has been working in the field for 20 years. [ … ] The important thing is, whether you attended a preventive program and whether it would have helped to avoid [an incident].”

(ID 15, personal communication, author’s translation)

The characteristics of an incident are particularly relevant for its classification and documentation. The first question that arises is whether there is immediate danger. Person 11 stated:

“A risk assessment should be carried out. Is this risk still present?”

(ID 11, personal communication, author’s translation)

The various forms of violence mentioned during the interviews are verbal, physical, sexualized, sexual, psychological, self-harm, spiritual, and cultural violence, as well as more specific subcategories. These include, for example, insults, shouting, discrimination, bullying, coercion, hitting, kicking, restraint, throwing objects, sexual harassment, and rape.

Interviewees also described the direction or level of violence also as relevant. Often it is not possible to clearly identify “perpetrator” and “victim”, as violent acts are often a reaction to previous triggers. Therefore, institutions distinguish between incidents at different levels, as Person 14 described:

“If you want to do it optimally, you would have to cover every constellation, “employee vs employee,” “manager vs employee,” “employee vs client,” “employee vs external person,” “client vs client,”, “client vs external person,” “self-aggression,” and “violence against objects”, i.e. eight constellations, in a violence protection concept and in the report form.”

(ID 14, personal communication, author’s translation)

The consequences of violence are an essential criterion for categorizing incidents. Person 1 stated:

“I can describe physical violence in more detail. [ … ] What I find much, much worse is that this verbal abuse leaves damage that I cannot see on the outside. And I find it incredibly difficult to portray that.”

(ID 1, personal communication, author’s translation

Visible injuries can be easily documented. This does not apply to mental health consequences. Person 2 suggested:

“Depending on how long ago this incident happened, I would hope that the question arises for me: To what extent am I dwelling about it, do thoughts caught me off guard, or am I constantly mulling over it subconsciously.”

(ID 2, personal communication, author’s translation)

Subjective perception was cited as the main criterion for determining severity. In addition, the criminal offense and the question of intent and ability are essential decision-making criteria. In settings with individuals with various disabilities or dementia, there is a risk of boundaries being crossed, which does not occur with malicious intent. Triggers can include a social-emotional age that leads to uninhibited behavior such as grabbing, pulling, and pinching. Delirium must also be considered in cases of doubt. Person 17 stated:

“Diagnostics are certainly helpful here. Disease or disorder profiles. Various syndromes that can also place demands on the framework conditions. For us, the social-emotional stage of development is also very important.”

ID 17, personal communication, author’s translation)

Person 15 stated:

“When we are in a crowded space with lots of people having severe disabilities, there is pinching, spitting, and biting. But they cannot control their behavior. This is a different situation compared to where people can control their behavior and may use it deliberately.”

ID 15, personal communication, author’s translation)

Finally, there are recurring incidents. Person 4 stated:

“A one-time occurrence must be dealt with differently than something that happens repeatedly. But it is not this factor alone that matters, but rather the severity of the violent incident.”

(ID 4, personal communication, author’s translation)

### 3.4 Deriving requirements for digital applications

Participants were specifically asked about requirements for an application that supports reporting, documenting and handling violent incidents in their respective institution.

First, data management, which allows secure data storage and handling according to the DSGVO/GDPR must be available and possible. The main feature should be to make it easy to report incidents. In addition, notifications (i.e., via smartphone, email or mobile app) has been mentioned several times. Person 1 stated:

“Could that perhaps be a mobile application for care professions that runs on my smartphone that I could download? [ … ] I could quickly report any incidents from home that I might not necessarily dare to report otherwise.”

(ID 1, personal communication, author’s translation)

Person 16 supplemented:

“That it can also be used on mobile devices. It should be user-friendly and collaborative. For example, the main user has an incident and everyone can provide more data, and that all perspectives are then brought together at the end.”

(ID 16, personal communication, author’s translation)

Another frequently mentioned requirement is “anonymous reporting”. Person 1 stated:

“Reporting incidents anonymously must be possible.”

(ID 1, personal communication, author’s translation)

Additionally, Person 5 mentioned:

“It has to be possible [to report anonymously] but this is always difficult. How am I supposed to contact and help that person?”

(ID 6, personal communication, author’s translation)

Interviewees also required simple language for the application. Person 7 stated:

“One thing is essential: simple language. We have people with significant language barriers. In my care home, we have between eight and twelve native languages. This means that people are completely overwhelmed by forms that are written in a very complicated language.”

(ID 7, personal communication, author’s translation)

Interviewees also mentioned an intuitive usability, which is described as self-explanatory, easy-to-use, simple design, requiring as little effort as possible. The majority of participants would also like to see a checklist-style report form that can be completed by ticking boxes or making selections, with as few text fields as possible.

Completing the report form is intended to initiate a process that includes all incident-related steps. This should be based on a clear, partially automated process structure (i.e., process model) that automatically informs individuals based on their role. In addition to documentation, these requirements also necessitate logging, and handling internal and external communication. Person 4 stated:

“Document all the steps we are already taking. From suspicion to immediate incident support, persons involved, actions already taken, include a reminder function to indicate whether the person who reported an incident has already received feedback. [ … ] The reminder functions for to-do tasks must be included. So, I would say, as in project management, tasks must be defined tight up to “process is complete.”

(ID 4, personal communication, author’s translation)

Person 8 further suggests:

“That means that data is collected somehow, a process is automatically initiated to determine what measures might be necessary and which specialist needs to be involved, and then the specialist service is automatically contacted and required data is provided.”

(ID 8, personal communication, author’s translation)

Person 14 added to this:

“When colleagues fill out the form, you don’t have to think about it anymore. Currently, I have to save this form, then send it to management, then send it to this and that department. Instead, it should be filled out and, depending on the location and severity level [ … ], it is directly forwarded to the right person after it is submitted. This simplifies the process even further. It pops up in the other places and then, of course, a procedure is executed.”

(ID 14, personal communication, author’s translation)

Status updates, assign responsibilities, send reminders and messages via the system or email, and suggest additional information and contacts for assistance should be included. In addition, validated instruments promoting self-reflection were mentioned several times as **Supplementary Material** for reporting employees. Also, involving the HR department and the automatic generation of accident reports for the (German) Employer’s Liability Insurance Association should be addressed. The final process phases discussed were the evaluation and the generation of an overview of key figures (or statistical analyses) for deriving preventive measures and inclusion in risk analysis.

## 4 Discussion

This work addresses the demand for developing a formal reporting process including a structure for immediate response and follow-up for violent incidents (10). Specifically, it focuses on elderly and special needs care institutions. The main functionality is providing digital documentation, spanning across all phases related to protection against violence. It further contributes to the possibility to statistical analyses for targeting the effectiveness and evidence of measures which is currently not possible (19).

It became clear that, when developing such an application, there should be different user groups. Especially secondary units should be able to receive and review data. This not only serves to improve internal coordination and enhance organizational and management interventions. The next task for the organizations is to precisely define which individuals are to play which roles in their processes. Direct communication with potential secondary units facilitates appropriate data preparation, ensuring that data can be processed properly. For organizations, the greatest benefit lies in improved communication among users, leading to better hazard assessment and risk analysis (4, 13). Making data immediately available, without media breaks, could also contribute to collaboration with external secondary units as a community intervention like recommended by Spencer et al. (10).

When talking about digital transformation, we usually mean an organizational and cultural transformation that integrates digital technology (20). The overarching obstacle here is to document and handle violent incidents. In doing so, we are responding to the call for systems to systematically record incidents of violence, including detailed documentation and monitoring (12, 13, 21). Our work revealed that a generalized process could be developed that is applicable to all institutions interviewed. Nevertheless, challenges remain. Initial processes for traditional, paper-based documentation and reporting of incidents are in place. However, there is still a lack of fundamental work regarding when incidents must be reported within organizations. This barrier of unclear institutional policies was also identified by Abreu et al. In addition to clear policies and a reporting system, the incorporation of a structured feedback process can facilitate reporting (5).

When asking about specific requirements for an assessment form during the interviews, a system that would allow objective, measurable, and transparent classification of violent incidents was proposed. As suggested by Caspi, this should be based on reported circumstances, causes, and triggers (22) and would ultimately contribute to the documentation and better handling of incidents of violence in the future.

The participants emphasized the importance of personalized assessment of all involved individuals. At the same time, they also mentioned the importance of quick and easy-to-use documentation. 12 of the participants called for a checklist-style form that would prevent lengthy clarification processes and documentation procedures involving long text-based entries, while still containing the most relevant information. Quantifiable factors that can be used in case handling were also mentioned. However, a more precise definition requires comprehensive discussions and perspectives. To gain a more concise picture, future work should examine involved actors at micro, meso, and macro levels, as well as the different phases of prevention, response, and recovery, as done by Vrablik et al. (4). The stakeholders to be considered during application development belong to different levels and require different data, which must be included in the reporting. The resulting process thus affects all three levels and phases. Related disciplines, such as psychology or law enforcement, could also provide important insights.

When evaluating these results, systematic and human factors must be carefully balanced. The introduction of new digital applications places new demands on employees (23). According to studies, technological innovations make caregivers feel more distant from the people they care for, which they perceive as a negative impact on their role (24). Violence protection must focus on human factors, such as care for victims and perpetrators. Considering the aforementioned phases of incidents, it became clear that the overall procedures are generally not properly defined across institutions and there is a lack of common sense how such incidents should be handled. This is also due to the missing definitions, like a uniform policy that specifies precise criteria for incidents of violence and determines when such incidents must be reported and documented, and, therefore, dealt with. Undoubtedly, there still remains an individual component, which is also in line with the statements by Schlup et al., according to which concepts must also take individual characteristics of caregivers, patients or clients, and institutions into account (25). Nevertheless, it could be helpful to view violence protection and case management as an overarching process within institutions. Continuous evaluations would reveal objective criteria to regularly improve these processes and, in turn, reduce stress in employees while simultaneously improving well-being at work.

When asked about application requirements, a list of features was compiled. The demand for system compatibility and interfaces to already existing software products are crucial. Depending on the very heterogeneous digitization strategies, technical equipment and (software) products in use within institutions this proves quite difficult. One controversial requirement extracted from the interviews is anonymous case reporting, where interviewees argued in diverging directions. No clear statements can be made about the effects of anonymous case reporting, and must be investigated more closely in future research. The requirements from the perspective of violence protection officers and managers provide valuable insights for an upcoming application development.

### 4.1 Implication for practice

This research provides concrete practical implications for future. A process-aware information system should enable to manage the overall process in one application. A process engine that is capable of executing such complex and knowledge- and communication intensive processes and providing the correct data to the person in charge at the right time is required (26). Such information systems help streamlining processes and automate the latter. For manual tasks (i.e., where humans are involved), they provide work-lists for employees to show currently available tasks and help structuring daily workload.

**Figure 2:**
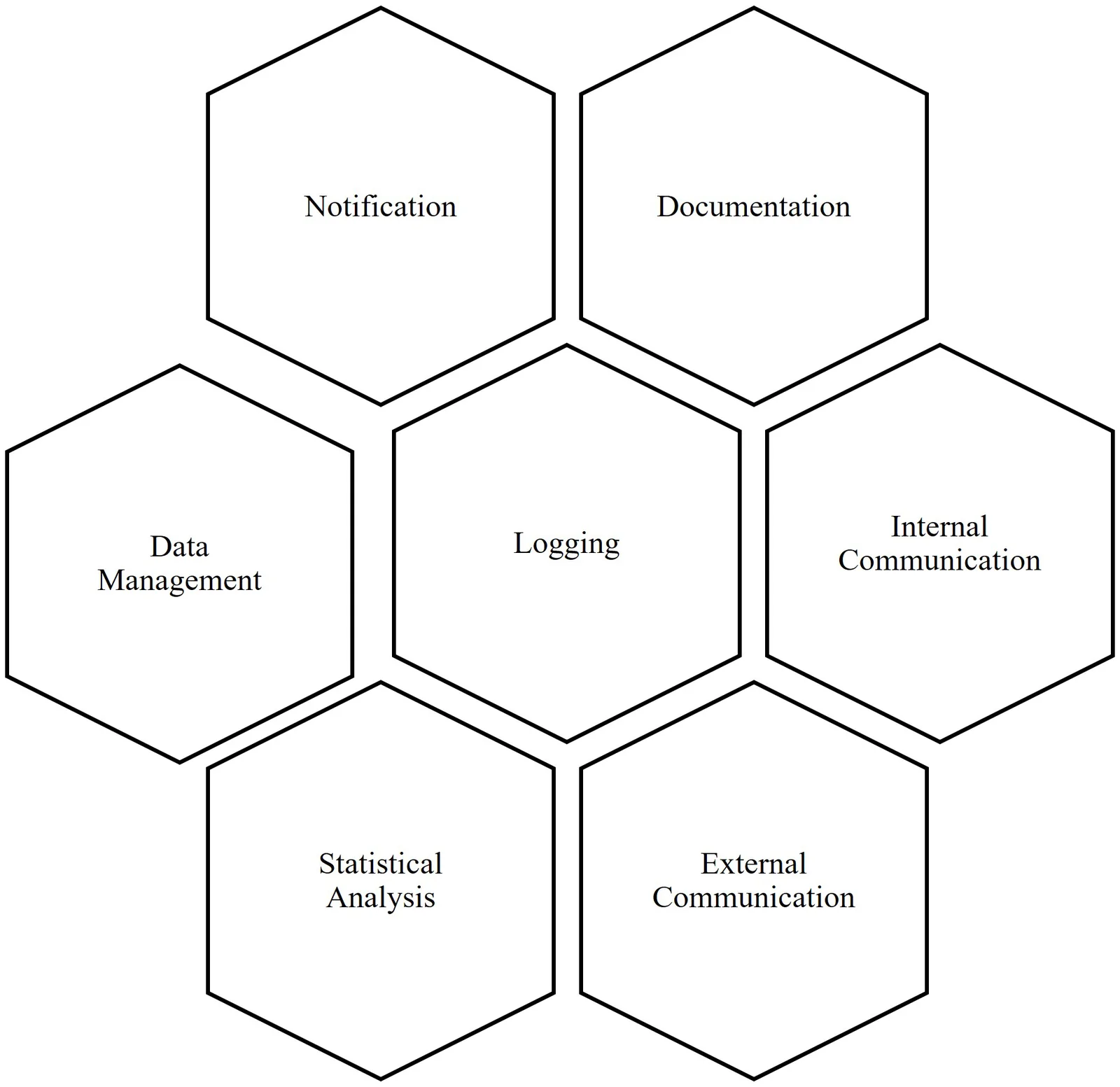
Most requested features for a digital application supporting documentation and reporting of violent incidents in elderly and special needs care.

**Figure 2** illustrates the mentioned requirements that could be digitally supported. The main feature of the application is to provide a complete logging. Case reporting involve filling out a checklist-like report form (i.e., questionnaire) for documentation, which triggers an immediate notification to the responsible person. An internal communication feature enables exchanging questions and continuously completing the report form. External communication is also necessary for case clarification and can be conducted digitally. It is important to properly handle different views on collected data based on the user roles within the process (i.e., incident reporter, witness, violence protection manager) and consider legal obligations (i.e., GDPR). The structured documentation can also be used for reports and statistical analysis with minimal effort. It became clear that the reporting and documentation process should be as simple as possible. Mobile reporting via various devices preferably via smartphone, was identified as the optimal solution. When relying on a process-aware information system, traceable and auditable logging is provided out-of-the-box.

### 4.2 Limitations

Limitations arise from the regional scope, the clear interests and motivations of all stakeholders, and the participants’ professional backgrounds. Interesting results could emerge from surveying other users. In addition, the existing findings have sparked discussion about various features such as anonymous reporting and incident assessment which should be further investigated. Furthermore, it was not possible to identify specific technical requirements, as the target group lacks the necessary technical expertise. Further research in this area should be conducted.

## 5 Conclusion

This research provides deep insights in the documentation and handling of violent incidents in elderly and special needs care institutions in southern Germany. Particular focus is on understanding and providing requirements for digitally supporting violence protection concepts. It became clear that there is certainly resistance to objectifying and structuring such a sensitive topic. Nevertheless, all study participants have recognized the importance of reporting, documenting and systematically analyzing incidents of violence and have taken the first steps toward doing so. Clear requirements for a digital application have been identified that would satisfy needs and demands from experts. This manuscript provides a foundation for practitioners and application developers to develop such an application. Implementing such an application will facilitate the structured reporting, handling, and evaluation of violent incidents and is demanded by domain experts. The data collected through such applications could be used to generate statistics that serve as a basis for developing concrete measures and recommendations for action. It became clear even during the study that defining objective criteria for classifying violence is particularly difficult. Further research is needed in this area.

## Data Availability

The raw data supporting the conclusions of this article will be made available by the authors, without undue reservation.

## Footnotes

1 https://gehba.de/; last accessed: 02/19/2026.

2 https://kompiga.de/; last accessed: 02/19/2026.
