# Supplementary material for: Rethinking violence prevention in elderly and special needs care: requirements for digital solutions
Source: Front Psychiatry. 2026 Aug 25;17:1843725. doi: 10.3389/fpsyt.2026.1843725 (PMC13551420; doi:10.3389/fpsyt.2026.1843725)
Supplement: Supplementary file 1 [file DataSheet1.pdf]

Supplementary Material

1 SUPPLEMENTARY DATA

1.1 Used quotes

Below are all the quotes used, presented in their original German form along with the corresponding English translation. The translation was done by one of the researchers and verified by the other. Any discrepancies were discussed taking the context into account.

Table S1: Original Quotes and English Translations

**Original Quote (German)**	**Translation (English)**
„Also entweder werde ich angerufen, also wenn ich vor Ort in der Einrichtung bin, oder ich kriege eine E-Mail. [. . .] Ich muss ja in dem Moment auch die Dokumentation lesen, die die Mitarbeiter ja niederschreiben und da hapert es manchmal schon. Also das ist so eine schwierige Stelle, wo ich viele Dinge nicht lesen kann. [. . .] Also das heißt, ich kann [. . .] das bei uns als Reiter oder als Möglichkeit anzukreuzen, hat aber der Mitarbeiter das nicht so gemacht, dann kann ich es aber auch nicht einsehen. Das heißt, erst in dem persönlichen Gespräch kann das erste rauskommen. Also das heißt, es kann sein, dass es zu einem Vorfall kommt, aber ich bekomme das erst Wochen oder Monate später mit. Also das ist schon, da ist eine Lücke da.“<br>(ID 13, persönliche Kommunikation)	„Either I get a call when I'm on site, or I receive an email. I also have to read the documentation [in the client file] that the employees wrote down. This is most likely quite difficult, as I often [haven't the access rights and] can't read all required information. If the employee doesn't check a field in the form, I can't see the information. That is revealed during personal conversation. An incident may occur, but I only find out about it weeks or months later. So that's definitely an issue. “<br>(ID 13, personal communication, author's translation)
„Dafür stehen uns Checklisten zur Verfügung [. . .]. Die kann man da aufrufen, die können die auch selber ausdrucken.“<br>(ID 7, persönliche Kommunikation)	„[...] we have checklists available [...]. You can access and print them out yourself.“<br>(ID 7, personal communication, author's translation)
„Ich gebe jedem zeitnah, also am besten direkt die Rückmeldung, ich habe dich gehört.“<br>(ID 2, persönliche Kommunikation)	„I give everyone feedback, preferably immediately, to let them know that I have heard them.“<br>(ID 2, personal communication, author's translation)

Table S1: Original Quotes and English Translations

**Original Quote (German)**	**Translation (English)**
„Das Krisenteam entscheidet dann, welche weiteren Personen hinzugezogen werden müssen. So wie ich es gerade gesagt habe, braucht es den Gewaltschutzbeauftragten der Einrichtung, braucht es die übergeordnete Führungskraft im Sinne einer Regionalleitung, muss es an die Geschäftsführung oder an den Vorstand hoch eskaliert werden.“ (ID 4, persönliche Kommunikation)	„The emergency response team decides which other people need to be involved in this case. Is it required to involve the violence protection officer? Is senior management required in the form of regional management? Does it need to be escalated to the management or the execution board? “ (ID 4, personal communication, author's translation)
„Toll wäre, wenn man das Ganze auch für die Dokumentation im Nachgang verwenden könnte. Wenn man die ganze Protokollierung da drin weiterführt. Wenn man da dann tatsächlich auch die Zuständigkeiten eintragen könnte, wer mit wem wie spricht, dann wäre das alles in einem. Man hätte den Datenschutz eingehalten, man müsste das nicht per Mail irgendwo hin und her schicken.“ (ID 15, persönliche Kommunikation)	„It would be great if you continue with the entire protocol there, if you could actually enter the responsibilities, who talks to whom and how, then everything would be in one place. Data protection would be complied with. You wouldn't have to send it back and forth via email. “ (IT 15, personal communication, author's translation)
„Es müssen klare Verantwortlichkeiten, wer was macht, verteilt werden. [. . .] Auch wie und was kommuniziert wird und was vielleicht nicht kommuniziert wird, muss klar sein. Es ist wichtig, dass die ganze Kommunikation nachvollziehbar ist.“ (ID 15, persönliche Kommunikation)	„Clear responsibilities must be assigned. [...] It is important that all communication is traceable.“ (ID 15, personal communication, author's translation)
„Es ist ein erstes, persönliches Gespräch, wo wir gemeinsam weitere Schritte gesprochen werden. Und dann ist es sehr individuell bezüglich des Vorfalls. Es besteht die Möglichkeit, ja, und also es kommt darauf an, was es ist. In einem Fall gibt es jetzt Supervisionen und in dem anderen gibt es, war die Beratung, ist ein sehr schneller Zugang zu probatorischen Sitzungen.“ (ID 2, persönliche Kommunikation)	„First, there is a direct conversation [with the reporting person] where we discuss the next steps. Then it depends very much on the individual case. [...] In one case, supervision is demanded, in another, counseling, and sometimes very quick access to probationary sessions is required [...].“ (ID 2, personal communication, author's tranlation)
„Die Fälle werden mir gemeldet. Und dann geht es um die Fallbearbeitung. Da steht immer als erstes der Schutz der Anvertrauten im Vordergrund oder Schutz der Mitarbeitenden, je nachdem.“ (ID 5, persönliche Kommunikation)	„Cases are directly reported to me. And then it's a matter of handling the cases. The highest priority is always protecting those entrusted to our care or our employees.“ (ID 5, personal communication, author's translation)

Table S1: Original Quotes and English Translations

Original Quote (German)	Translation (English)
„Das müsste [. . .] auch die Täteraspekte berücksichtigen, weil wir meistens für den Täter ja auch verantwortlich sind.“ (ID 11, persönliche Kommunikation)	„The perpetrator's perspective must also be taken into account, because we are usually responsible for the perpetrator as well.“ (ID 11, personal communication, author's translation)
„Die sind ja oft auch in der Not und oftmals nicht bewusst, sondern sind halt in ihrem Rahmen, in ihren Möglichkeiten manchmal auch übers Ziel raus.“ (ID 11, persönliche Kommunikation)	„They [perpetrators] are often in distress and unaware, but simply acting within their own boundaries and possibilities, sometimes going too far“ (ID 11, personal communication, author's translation)
„Es wird immer der Betroffene angesprochen [. . .] Das ist immer ein schwieriges Thema, weil da ist der Gewaltvorwurf, auf der anderen Seite kann es immer auch Verleumdung sein. Es ist wichtig die rechtliche Seite dabei nicht außenvor zu lassen.“ (ID 6, persönliche Kommunikation)	„We always talk to those affected. [...] Legal aspects must also be examined. This may be difficult, because on the one hand there is the accusation of violence, but on the other hand it could also be defamation“ (ID 6, personal communication, author's translation)
„Und wir haben, wenn es um Minderjährige geht, also um nicht Volljährige, auch nach gewisse Kriterien die Pflicht, Jugendämter zu informieren.“ (ID 16, persönliche Kommunikation)	„When it comes to minors, i.e., those who are not of legal age, we also have a duty to inform the youth welfare office according to certain criteria.“ (ID 16, personal communication, author's translation)
„Wir machen jährlich auch Evaluationstermine für die Statistik und die Bewertung der Maßnahmen für die Gesamteinrichtung mit allen Geschäftsleitungen, den Vorständen, Facharzt für Psychiatrie, Fachdiensten, Deeskalationstrainerinnen, um dann nochmal global Maßnahmen zu entwickeln.“ (ID 18, persönliche Kommunikation)	„We also hold annual evaluation meetings with all management teams to review statistics and assess measures for the entire institution [...], with the aim to develop new measures at the corporate level.“ (ID 18, personal communication, author's translation)
„Wir schauen zwei, drei Monate, nachdem das so weit abgearbeitet ist, nochmal den Prozess an. - Und nicht, was ist jetzt der Stand inhaltlich, sondern wie lief die Meldekette? - Wo gibt es irgendwie Probleme, um auch den Prozess regelmäßig zu verbessern?“ (ID 15, persönliche Kommunikation)	„We review each case two to three months after it has been resolved. We are not interested in the current status, but rather at how the reporting process worked. Can we identify any problems and improve the process?“ (ID 15, personal communication, author's translation)

Table S1: Original Quotes and English Translations

Original Quote (German)	Translation (English)
„Aber es sollte angeschaut werden, also wie ist die räumliche Situation? Wie ist die personelle Situation? Wie ist die fachliche Kompetenz der Mitarbeitenden? Wie werden die eingeführt? Wie werden die eingearbeitet? Wie werden die vom Fachdienst begleitet? Das muss man mit anschauen und man muss auch anschauen, was ist drum herum gerade los? Also Weihnachten, Weihnachten oder irgendwelche großen Jahresfeste, wo die Erwartung und die Spannung der BewohnerInnen steigt. [. . .] Die Anspannung steigt und damit auch unter Umständen die Möglichkeit, dass irgendwelche Situationen eskalieren.“ (ID 18, persönliche Kommunikation)	„It is important to consider the spatial situation. What is the staffing situation? What is the professional competence of the employees involved in an incident? How are they being trained? How are they being supported by the specialist service? Then you also have to take into account what is currently happening in their environment. For example, Christmas or any other major annual celebrations, when the residents' expectations and excitement increase. [...] Tension rises, and with it the possibility that certain situations will escalate.“ (ID 18, personal communication, author's translation)
„Wir finden zu wenig offen. Es ist da. Und alle, wenn man auch ins Gespräch geht, denken alle, ja wir sind doch gut. Wir machen doch schon viel. Es würde eine Offenheit bestehen. Und plötzlich stelle ich in dem Gespräch fest, ah, hier findet gerade eine Selbstreflexion statt, dass die Leute sich fragen, ja Mensch, aber da übe ja auch ich Gewalt aus.“ (ID 2, persönliche Kommunikation)	„[The topic of violence] is still not discussed openly enough. It's there. When you talk to people, they all think, yes, we're good. We're already doing a lot. And suddenly, during the conversation, I realize, ah, there's some self-reflection going on here, that people are asking themselves, yes, but I'm also applying violence“ (ID2, personal communication, author's translation)
„Also wenn ich mir meinen Traumata nicht bewusst bin, dann werde ich natürlich ständig verletzt und erfahre Gewalt oder es kommt so eine Eskalation. Und eben wenn mir das bewusst ist, dann kann ich mich auch dementsprechend auch davor schützen.“ (ID 13, persönliche Kommunikation)	„If I am not aware of my trauma, then of course I will constantly be hurt and experience violence, or things will escalate. And if I am aware, I can protect myself accordingly.“ (ID 13, personal communication, author's translation)
„Also ich glaube, es ist ein Unterschied, ob ein Schüler kommt, der vielleicht gerade Praktikant ist, oder zu einer erfahrenen Kraft, die seit 20 Jahren in dem Bereich arbeitet und die eine oder andere Situation einfach anders einschätzt oder schon präventiv anders arbeitet, so dass vielleicht gar nicht die Eskalation entsteht. [. . .] Wichtig wäre auch ob man bei einer Präventivveranstaltung war, ob da was geholfen hätte.“ (ID 15, persönliche Kommunikation)	„It makes a difference, if it is a student or an experienced professional who has been working in the field for 20 years. [...] The important thing is, whether you attended a preventive program and whether it would have helped to avoid [an incident“ (ID 15, personal communication, author's translation)

Table S1: Original Quotes and English Translations

Original Quote (German)	Translation (English)
„Und eine Risikobeurteilung muss bestehen. Besteht die Gefährdungslage weiter?“ (ID 11, persönliche Kommunikation)	„A risk assessment should be carried out. Is this risk still present.“ (ID 11, personal communication, author's translation)
„Das heißt, wenn man es optimal machen möchte in der Erfassung, müsste man jede Konstellation erfassen, also Mitarbeiter, Klient, Mitarbeiter, Mitarbeiter, Klient, Mitarbeiter, Klient, Klient. Dann könnte man auch noch erfassen, Führungskraft, Mitarbeiter, externe Person, Klient, externe Person, Mitarbeiter, Gewalt gegen sich selber Gewalt gegen Gegenstände.“ (ID 14, persönliche Kommunikation)	„If you want to do it optimally, you would have to cover every constellation, "employee vs employee," "manager vs employee," "employee vs client," "employee vs external person," "client vs client", "client vs external person", "self-aggression", and "violence against objects", i.e. eight constellations in a violence protection concept and in the report form. “ (ID 14, personal communication, author's translation)
„Und physische Gewalt kann ich ja noch mal beschreiben. [. . .]Was ich viel, viel schlimmer finde, sind dass diese, diese Wortgewalt, die ausgeübt wird, die hinterlässt da Schäden, die kann ich nicht nach außen sehen. Und das, also das abzubilden, das finde ich unheimlich schwierig.“ (ID 1, persönliche Kommunikation)	„I can describe physical violence in more detail. [...] What I find much, much worse is that this verbal abuse leaves damage that I cannot see on the outside. And I find it incredibly difficult to portray that“ (ID 1, personal communication, author's translation)
„Und abhängig davon, wie lange es her ist, wenn es länger zurückliegt, würde ich mir wünschen, dass die Frage, wie oft muss ich daran denken, denke ich daran unvorbereitet oder grübele ich da unbewusst permanent darüber nach.“ (ID 2, persönliche Kommunikation)	„Depending on how long ago this incident happened, I would hope that the question arises for me: To what extent am I dwelling about it, do thoughts caught me off guard, or am I constantly mulling over it subconsciously“ (ID 2, personal communication, author's translation)
„Dann sind sicherlich Diagnostiken hilfreich. Krankheits- oder Störungsbilder. Verschiedene Syndrome, die auch Anforderungen an die Rahmenbedingungen stellen können. Bei uns ist noch ganz wichtig der sozio-emotionale Entwicklungsstand.“ (ID 17, persönliche Kommunikation)	„Diagnostics are certainly helpful here. Disease or disorder profiles. Various syndromes that can also place demands on the framework conditions. For us, the social-emotional stage of development is also very important.“ (ID 17, personal communication, author's translation)

Table S1: Original Quotes and English Translations

**Original Quote (German)**	**Translation (English)**
„Wenn wir zum Beispiel in überfüllten Bereichen sind. Bei unserer Einrichtung gibt es Bereiche, wo Schwerst-Mehrfach-Behinderte sind, wo gezwickt, gespuckt, gebissen wird, die das aber gar nicht steuern können. Das ist eine andere Situation als in Bereichen, wo Menschen das steuern können, wo das vielleicht gezielt eingesetzt wird.“<br>(ID 15, persönliche Kommunikation)	„When we are in a crowded space with lots of people having severe disabilities, there is pinching, spitting, and biting. But they cannot control their behavior. This is a different situation compared to where people can control their behavior and may use it deliberately.“<br>(ID 15, personal communication, author’s translation)
„Ich glaube, dass man mit einer einmaligen Sache anders umgehen muss wie mit etwas, was mehrfach vorkommt, aber nicht dieser Faktor allein, sondern im Zusammenhang mit der Schwere des Gewaltvorfalls.“<br>(ID 4, persönliche Kommunikation)	„A one-time occurrence must be dealt with differently than something that happens repeatedly. But it is not this factor alone that matters, but rather the severity of the violent incident“<br>(ID 4, personal communication, author’s translation)
„Ich habe die ganze Zeit schon spuckt mir das im Hinterkopf, ob das vielleicht eine App auf dem Smartphone, die ich mir runterziehen könnte für Pflegeberufe. [. . .] Weil die App wäre natürlich super, weil da kann ich einfach auch schnell von zu Hause aus noch irgendeinen Vorfall melden, wo ich mich vielleicht sonst nicht unbedingt trauen würde.“<br>(ID 1, persönliche Kommunikation)	„Could that perhaps be a mobile application for care professions that runs on my smartphone that I could download? [...] I could quickly report any incidents from home that I might not necessarily dare to report otherwise.“<br>(ID 1, personal communication, author’s translation)
„Dass sie vielleicht auch mobil handhabbar ist. Also dass sie einfach nutzerfreundlich ist. Und dass sie vielleicht auch vernetzbar ist zum Beispiel. Dass die fünf Hauptakteure, von denen ich gesprochen habe, beide so ein Ding haben und jeder kann es füttern. dass das dann zusammengeführt wird.“<br>(ID 16, persönliche Kommunikation)	„That it can also be used on mobile devices. It should be user-friendly and collaborative. For example, the main user has an incident and everyone can provide more data, and that all perspectives are then brought together at the end.“<br>(ID 16, personal communication, author’s translation)
„Eine anonymisierte Meldung muss möglich sein“<br>(ID 1, persönliche Kommunikation)	„Reporting incidents anonymously must be possible.“<br>(ID 1, personal communication, author’s translation)
„Es [anonyme Meldung] muss möglich sein. Wenn es völlig anonym ist, dann ist es immer schwierig. Wie kann ich Kontakt aufnehmen und demjenigen dann helfen?“<br>(ID 6, persönliche Kommunikation)	„It has to be possible [to report anonymously] but this is always difficult. How am I supposed to contact and help that person?“<br>(ID 6, personal communication, author’s translation)

Table S1: Original Quotes and English Translations

Original Quote (German)	Translation (English)
„Das eine muss sein, tatsächlich leichte Sprache. Wir haben Leute mit einer hohen Sprachbarriere. In meinem Pflegeheim hatten wir, als ich dann noch tätig war, zwischen acht und zwölf Muttersprachen. Das heißt, die Leute sind mit einem sehr kompliziert ausgedrückten Bogen heillos überfordert.“ (ID 7, persönliche Kommunikation)	„One thing is essential: simple language. We have people with significant language barriers. In my care home, we have between eight and twelve native languages. This means that people are completely overwhelmed by forms that are written in a very complicated language.“ (ID 7, personal communication, author’s translation)
„Sie müsste alle Schritte abbilden, die wir jetzt auch schon gehen. Also von Verdacht über akutes Ereignis, beteiligte Personen, Schritte, die eingeleitet werden, auch eine Erinnerungsfunktion, ob der Person, die gemeldet hat, schon eine Nachricht gegeben worden ist [. . .], die Erinnerungsfunktionen für To-Do-Schritte drin hat. Also ich sag mal wie im Projektmanagement, das Aufgaben definiert werden bis hin Prozess ist abgeschlossen.“ (ID4, persönliche Kommunikation)	„Document all the steps we are already taking. From suspicion to immediate incident support, persons involved, actions already taken, include a reminder function to indicate whether the personwho reported an incident has already received feedback. [...] The reminder functions for to-do tasks must be included. So, I would say, as in project management, tasks must be defined tight up to ’process is complete’.“ (ID 4, personal communication, author’s translation)
„Also das heißt, es werden irgendwie Erfassungsdaten gesammelt, es geht automatisch ein Prozess los, was könnte als Maßnahme notwendig sein, welcher Fachdienst müsste einbezogen werden und automatisch wird dann quasi der Fachdienst kontaktiert und hat dann alle Informationen.“ (ID 8, persönliche Kommunikation)	„That means that data is collected somehow, a process is automatically initiated to determine what measures might be necessary and which specialist needs to be involved, and then the specialist service is automatically contacted and required data is provided.“ (ID 8, personal communication, author’s translation)
„Dass wenn Kollegen das [Formular] ausfüllen, dass man nicht noch dran denken muss, also jetzt muss ich dieses Formular noch abspeichern, dann muss ich das also der Leitung zuschicken. Dann muss ich es noch an die Stelle schicken, dann muss ich es vielleicht noch dahin schicken, sondern es wird ausgefüllt. Und je nachdem, welcher Bereich oben angeklickt wird und wie schwer der Vorfall ist geht es direkt an die Stellen. Dann wird der Prozess noch mal vereinfacht, es ploppt an den anderen Stellen auf und dann braucht es auch dann natürlich wieder einen klaren Ablauf.“ (ID 14, persönliche Kommunikation)	„When colleagues fill out the form, you don’t have to think about it anymore. Currently, I have to save this form, then send it to management, then send it to this and that department. Instead, it should be filled out and, depending on the location and severity level [...], it is directly forwarded to the right person after it is submitted. This simplifies the process even further. It pops up in the other places and then, of course, a procedure is executed.“ (ID 14, personal communication, author’s translation)
